# Clinico-radiological and molecular characterization of a child with ring chromosome 2 presenting growth failure, microcephaly, kidney and brain malformations

**DOI:** 10.1186/s13039-015-0121-z

**Published:** 2015-03-05

**Authors:** Mariasavina Severino, Andrea Accogli, Giorgio Gimelli, Andrea Rossi, Svetlana Kotzeva, Maja Di Rocco, Patrizia Ronchetto, Cristina Cuoco, Elisa Tassano

**Affiliations:** Neuroradiology Unit, Istituto Giannina Gaslini, Genoa, Italy; Pediatric Pulmonology and Allergy Unit, Istituto Giannina Gaslini, Genoa, Italy; Laboratorio di Citogenetica, Istituto Giannina Gaslini, Genoa, Italy; Anesthesiology Unit, Istituto Giannina Gaslini, Genoa, Italy; Pediatria II, Istituto Giannina Gaslini, Genoa, Italy

**Keywords:** Array-CGH, 2p25.3 deletion, 2q37.3 deletion, Ring chromosome 2, Brain MRI, Diffusion tensor imaging

## Abstract

**Background:**

Ring chromosome 2 is a rare constitutional abnormality that generally occurs *de novo*. About 14 cases have been described to date, but the vast majority of papers report exclusively conventional cytogenetic investigations and only two have been characterized by array-CGH.

**Results:**

Here we describe the clinical, neuroradiological, and molecular features of a 5-year-old boy harbouring a ring chromosome 2 presenting with severe growth failure, facial and bone dysmorphisms, microcephaly, and renal malformation. Brain MR with diffusion tensor imaging revealed simplified cortical gyration, pontine hypoplasia, and abnormally thick posterior corpus callosum, suggesting an underlying axonal guidance defect. Cytogenetic investigations showed a karyotype with a ring chromosome 2 and FISH analysis with subtelomeric probes revealed the absence of signals on both arms. These results were confirmed by array-CGH showing terminal deletions on 2p25.3 (~439 kb) and 2q37.3 (~3.4 Mb).

**Conclusions:**

Our report describes a new patient with a ring chromosome 2 completely characterised by array-CGH providing additional information useful not only to study genotype-phenotype correlation but also to validate the role of already reported candidate genes and to suggest novel ones which could improve our understanding of the clinical features associated with ring chromosome 2.

**Electronic supplementary material:**

The online version of this article (doi:10.1186/s13039-015-0121-z) contains supplementary material, which is available to authorized users.

## Background

Ring chromosomes result from one or two distal breaks followed by fusion of the broken ends and loss of the acentric fragments, but they can also be formed by fusion of subtelomeric sequences without apparent loss of genetic material. The ring chromosome phenotype is characterized by growth failure and intrauterine growth retardation (IUGR). According to Cote et al. [1] and Kosztolanyi et al. [2], which defined the “Ring syndrome”, suggested that the common growth deficiency, observed across many patients with diverse ring chromosomes, was due to mitotic instability and tissue-specific mosaicism. Recently, FISH and CGH studies demonstrated that, in most cases, a cryptic deletion is the cause of the phenotypic abnormalities in apparently intact rings.

Here, we report on a boy with a ring chromosome 2 presenting growth failure, microcephaly, dysmorphic features, L-shaped kidney, and brain malformations studied with brain MRI and diffusion tensor imaging.

## Case presentation

The patient is a 5-year-old boy, only child of healthy non-consanguineous parents. He was born to a 28-year-old mother whose pregnancy was complicated by IUGR and oligohydramnios, requiring caesarean section at 35 weeks of gestation. The Apgar score was 9 and 10 at 1 and 5 minutes, respectively. Physical examination at birth revealed left clubfoot (congenital talipes equinovarus) that was treated with a plaster cast. Birth weight was 1740 g (<3^rd^ centile), length 40.5 cm (<3^rd^ centile) and head circumference 29.5 cm (<3^rd^ centile). He developed mild respiratory distress requiring continuous positive airway pressure (nCPAP) for 72 hours. Echocardiogram depicted patent ductus arteriosus that disappeared spontaneously at follow-up. Abdominal ultrasound revealed crossed renal ectopia resulting in an L-shaped kidney (the left crossed kidney assumed a transverse position in the pelvis), while brain ultrasound showed incomplete opercularization.

**Figure 1:**
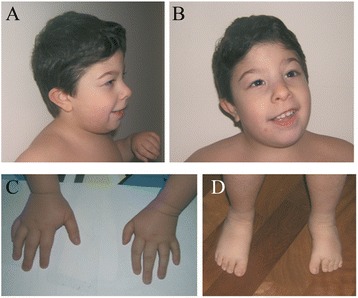
**Patient at 5 years of age. (A)** Lateral and **(B)** frontal views of the face of the boy at 5 years of age show medial eyebrow flare, wide nasal bridge, hypertelorism, epicanthal folds, broad nasal tip with prominent columella, long philtrum, thin upper lip, short neck. Hands **(C)** and feet **(D)** pictures demonstrate small hands and feet with clinodactyly and brachydactyly and left clubfoot.

At the age of 2 years, weight and length were well below the 3^rd^ centile and head circumference was 4 SD below the mean. Dysmorphic features (Figure 1A,B) included round face, short forehead, medial eyebrow flare, wide nasal bridge, hypertelorism, epicanthal folds, broad nasal tip with prominent columella, long philtrum, thin upper lip, mild protruding ears, short neck, small hands with brachydactyly and clinodactyly of II left digit and V digit bilaterally, feet with broad big toes, brachydactyly, and left clubfoot (Figure 1C,D). Neurological evaluation revealed mild developmental delay (he had a developmental age of 15 months at the age of 2 years in according to Griffith scale) and global hypotonia. He walked with support at 14 months and emitted vocalizations and babbling at the age of 19 months. The ophthalmologic evaluation was negative.

**Figure 2:**
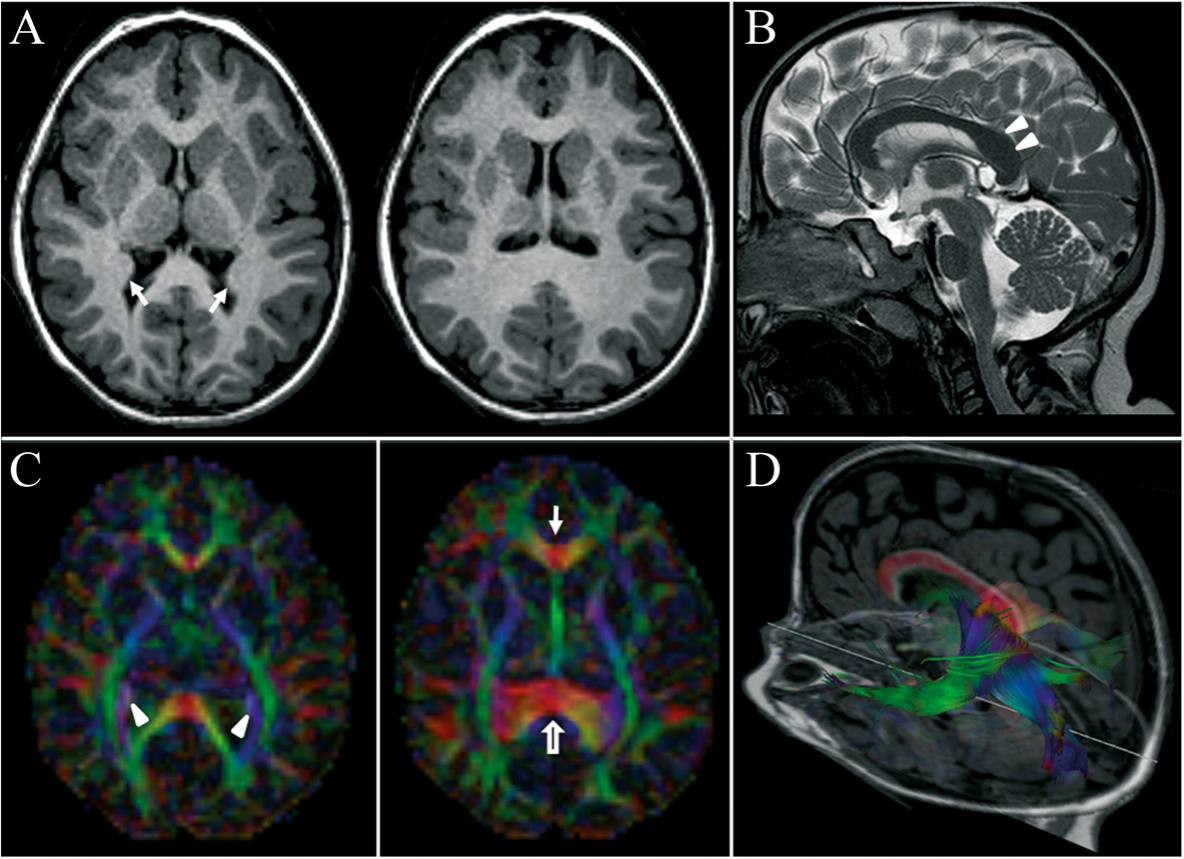
**Brain MRI and DTI at 2 years of age. (A)** Axial T1-weighted images reveal microcephaly with simplified cortical gyration and underdevelopment of the frontal lobes. Note the inward deformation of the lateral walls of the trigones (arrows). **(B)** Sagittal T2-weighted image shows pontine hypoplasia and dysmorphic corpus callosum with enlarged splenium (arrowheads). **(C)** Corresponding axial fractional anisotropy (FA)-weighted colour directional maps confirm the disproportion between the genu (arrow) and the splenium (empty arrow) of the corpus callosum. Note the presence of abnormally thickened fiber bundles with superior-to-inferior direction, located between the optic radiations and the lateral ventricles (arrowheads). The fibers are color-coded by direction: red is left-to-right (and vice versa), green is anterior-to-posterior (and vice versa), blue is superior-to-inferior (and vice versa). **(D)** DTT, oblique view, shows the course of the splenium of the corpus callosum superimposed on sagittal and axial T1-weighted images fused with FA-weighted colour directional map. The inward deformation of the lateral walls of the trigones is due to an abnormally thick tapetum. The tapetum is made of fibers of the corpus callosum connecting the inferior temporal lobes.

At the age of 2 years, brain MRI revealed microcephaly with simplified cortical gyration, bilateral hippocampal malrotation, and pontine hypoplasia. The corpus callosum appeared abnormally thickened at the level of the splenium and the lateral ventricles were dysmorphic (Figure 2A,B). Diffusion tensor imaging (DTI) and tractography (DTT) confirmed the disproportion between the anterior and posterior segments of the corpus callosum with an abnormally thick splenium. No ectopic callosal bundles were found. Interestingly, the tapetum was hyperplasic and caused a peculiar inward deformation of the lateral walls of the ventricular trigones (Figure 2C,D).

## Results

**Figure 3:**
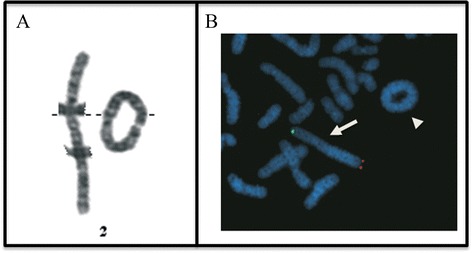
**Karyotype and FISH analyses. (A)**: Partial G-banded karyotype of peripheral blood lymphocytes showing a normal chromosome 2 and ring chromosome 2. **(B)** Metaphase FISH analysis shows absence of the 2p subtelomeric probe signal (spectrum green) and 2q subtelomeric probe signal (spectrum red) on the ring chromosome 2.

Cytogenetic analysis performed on lymphocytes from peripheral blood showed a large ring chromosome 2 with a preponderance of cells with the ring (92 metaphases) next to a minority of cells without the ring (8 metaphases). (Figure 3A). The karyotype was mos 46,XY, r(2)(p25.3q37.3) [92]/46,XY[8]. Karyotypes of the parents were normal.

**Figure 4:**
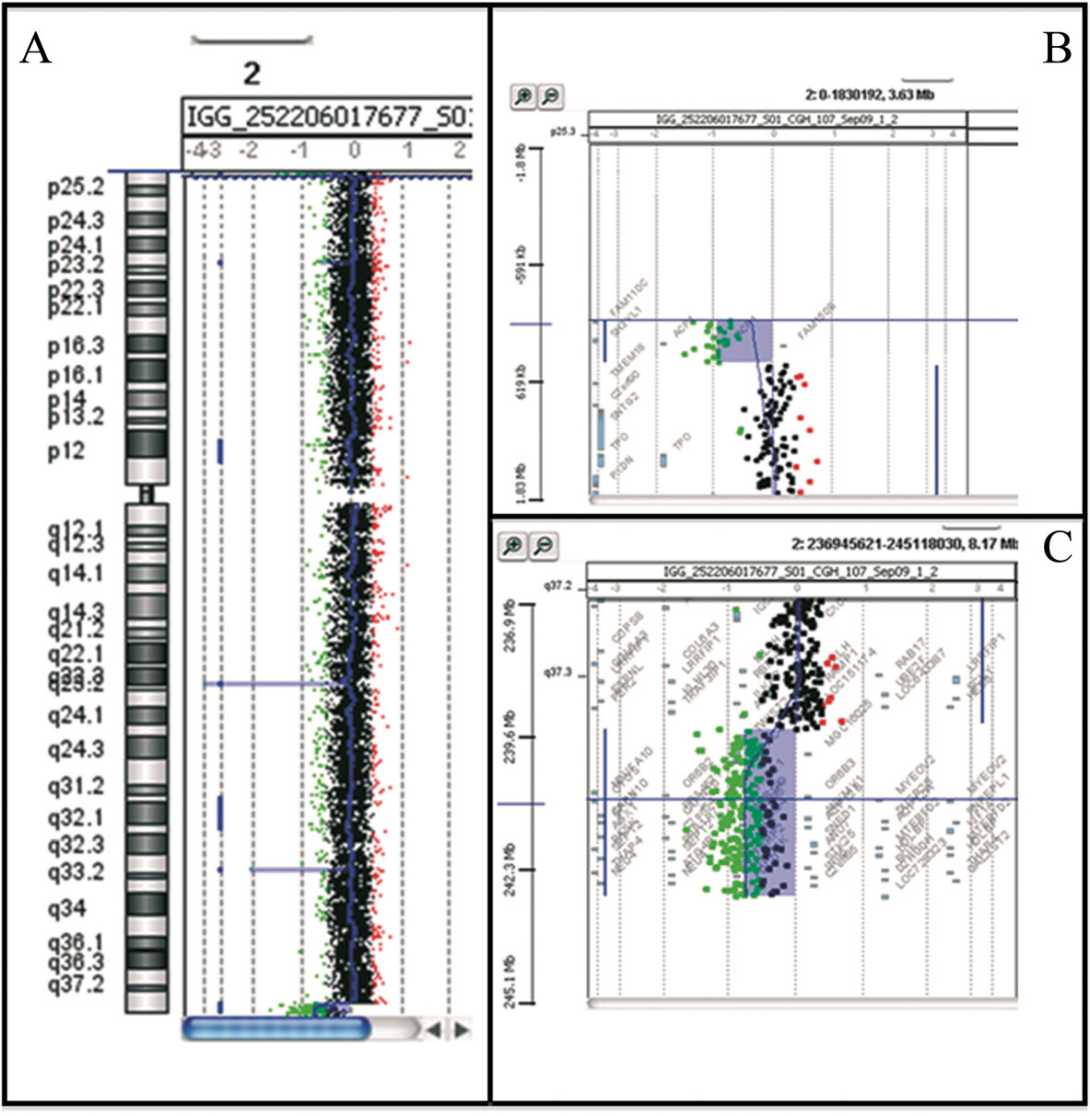
**Results of array**-**CGH analysis. A)** Chromosomal view. **B)** Zoom in view of short arm of the ring chromosome 2 shows a ~470 kb deletion at 2 pter-p25.3 (chr2:0–469,973) (NCBI build 37). **C)** Zoom in view of long arm of the ring chromosome 2 shows a 3.4 Mb deletion at 2q37.3-qter(chr2:239,636,228-243,041,364). Array-CGH results were described as: arr 2pterp25.3(chr2:30,341-469,973)x1, 2q37.3qter(chr2:239,636,228-243,041,364)x1 (ISCN 2013).

Array-CGH analysis revealed a deletion of ~470 kb at the distal region of the short arm of the ring chromosome 2 pter-p25.3 (chr2:0–469,973) (NCBI build 37) and a large deletion of ~3.4 Mb (chr2:239,636,228-243,041,364) at the distal long arm of the ring chromosome at bands 2q37.3-qter [arr 2pterp25.3(30,341-469,973)x1, 2q37.3qter(239,636,228-243,041,364)x1] (Figure 4). Array-CGH was confirmed by FISH (Figure 3B). The deleted 2p25.3 region contains only four genes (*FAM110C*, *SH3YL1*, *ACP1*, *FAM150B*). The deleted 2q37.3-qter region contains 27 OMIM genes (Additional file 1: Table S1).

## Discussion

Ring chromosomes are rare and present a classical phenotype characterized by minor dysmorphic facial features and mental retardation. According to Cote et al. [1] and Kosztolanyi, [2], the “Ring syndrome” is essentially characterised by intrauterine growth retardation (IUGR), and failure to thrive, which can be the sole major physical abnormalities. The common growth deficiency observed in many patients with different ring chromosomes suggested that this phenomenon is due to mitotic instability of the ring chromosome and tissue-specific mosaicism [2].

The application of FISH and, more recently, of array-CGH demonstrated that, in most cases, a cryptic deletion could determine phenotypic abnormalities in apparently intact rings. Our patient carried a ring chromosome 2 with a 2pter-p25.3 deleted region of ~470 kb and a concomitant deleted region of ~3.405 Mb at bands 2q37.3-qter. This ring chromosome appeared to be a mosaic with a proven *de novo* origin.

Ring chromosome 2 is very rare and has been reported in just 13 cases, but only our case and the two reported respectively by López-Uriarte et al. and Chen et al. were analysed by array-CGH [1,3-12].

**Figure 5:**
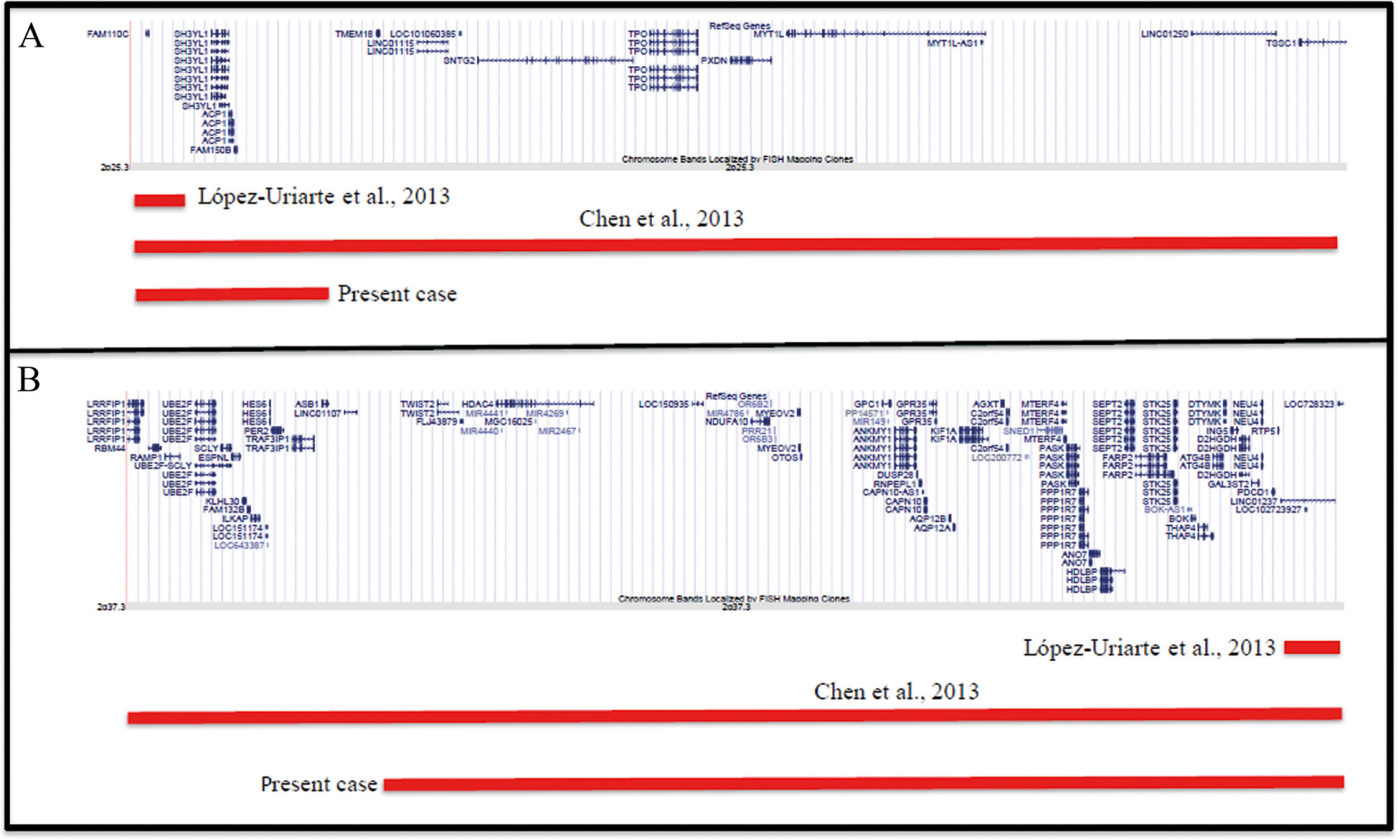
**Overview of the deleted regions 2pter-p25.3 A) and 2q37.3-qter B) of the ring chromosome.** Gene content according to the UCSC Genome Browser [GRCh37/hg19 assembly]. The bars indicate the deleted region (red) in our patient and the deleted regions in patients reported by López-Uriarte et al. [12] and Chen et al. [11].

The ring chromosome described by López-Uriarte et al. [12] presented a deleted region of 139 kb at 2p25.3 including only *FAM110C* gene, while the deleted region of ~147 kb at 2q37.3 contained no coding genes. The patient was a 10-month-old male with failure to thrive, microcephaly, and minor dysmorphic features. Differently, the ring 2 reported by Chen et al. [11] showed a 3.3 Mb deletion at chromosome band 2p25.3 and a 4.4 Mb deletion at chromosome band 2q37.3. The deleted 2p25.3 region contained 8 OMIM genes (*FAM110C*, *ACP1*, *TMEM18*, *SNTG2*, *TPO*, *PXDN*, *MYT1L*, and *TSSC1*), while the deleted 2q37.3 region, in addition to the genes present in our deletion, contained the following OMIM genes: *LRRFIP1*, *RAMP1*, *SCLY*, *HES6*, *PER2*, *TRAF3IP*1, and *ASB*1 (Figure 5).

In our opinion, the patient described by Lopez-Uriarte et al. [12] showed the features of the so-called “Ring syndrome”, characterised by growth failure, microcephaly, and minor dysmorphic features. Some features of the “Ring syndrome”, as IUGR and failure to thrive, were present also in our patient, who however showed other manifestations that could be the result of the deleted regions of ring chromosome 2. In fact, our case has a 3.4 Mb microdeletion in 2q37.3. It is known that 2q37 deletions have been associated to the Brachydactyly-Mental Retardation syndrome (MIM 600430), a contiguous gene syndrome characterized by haploinsufficiency or heterozygous mutations in the *HDAC4* gene. The human *HDAC4* gene encodes a chromatin-remodelling factor, histone deacetylase 4, which cooperatively regulates gene expressions with other transcription factors in the physiological process of development and differentiation of various tissues [13,14].

The main phenotypic features, reviewed by Leroy et al. [15], include mild-moderate developmental delay/intellectual disability, brachymetaphalangy of digits 3–5 (often digit 4 alone) (>50%), short stature, obesity, hypotonia, characteristic facial appearance, autism or autism spectrum disorder (30%), joint hypermobility/dislocation, and scoliosis. Other findings include seizures (20-35%), congenital heart disease, central nervous system (CNS) abnormalities, umbilical/inguinal hernia, tracheomalacia, situs abnormalities, gastrointestinal abnormalities, and renal malformations [16]. Likewise, our patient showed developmental delay, brachydactyly, renal, and brain anomalies. However, three individuals with haploinsufficiency of *HDAC4* showing brachydactyly type E, non-dysmorphic facial features, and normal intelligence have been reported [17]. The authors speculated that haploinsufficiency of *HDAC4* is not sufficient to cause intellectual disability (ID) in all affected individuals, but ID is likely due to haploinsufficiency of multiple genes.

The deleted region in our patient contained also *KIF1A* gene. *KIF1A* belongs to the kinesin 3 family, a large superfamily of molecular motors using microtubules as a “rail” to transport cargo along and chemical energy of ATP to drive conformational changes that generate motile force. Active transport of proteins along directional cytoskeletal filaments is fundamental for neuronal function and survival, because most of the proteins required in the axon and nerve terminals need to be transported from the cell body [18]. Homozygous or compound heterozygous mutation in the *KIF1A* is implicated in mental retardation, autosomal dominant 9 (MIM 614255), neuropathy, hereditary sensory, type IIC (MIM 614213) and spastic paraplegia 30, autosomal recessive (MIM 610357).

Interestingly, our patient presented brain anomalies such as microcephaly with simplified cortical gyration associated with pontine hypoplasia, hippocampal malrotation and callosal abnormalities. In particular, the corpus callosum was dysmorphic with an abnormally thick splenium. DTI revealed the presence of a hypertrophic splenium suggesting an underlying axonal guidance defect altering the normal corpus callosum development. Intriguingly, another gene included in the deleted region of the present case, *FARP2*, encodes for a GTPase involved in neurite growth and axonal guidance. In particular, FARP2 is an immediate downstream signalling molecule of the Sema3A receptor complex that mediates repulsion of outgrowing axons and suppression of neuronal adhesion [19].

This peculiar combination of brain malformations has never been described in patients with ring chromosome 2 or 2q37.3 syndrome. This suggests that other pathogenic genes like *TWIST2*, *NDUFA10*, *SEPT2*, *GAL3ST2*, and *NEU4*, present in the deleted region, could have contributed to the overall phenotype of our patient.

Actually, the genes included in the deleted region 2pter-p25.3 (*FAM110C*, *SH3YL1*, *ACP1*, *FAM150B*) did not appear to be causative of the phenotype of our patient, but we cannot exclude this hypothesis.

## Conclusions

In conclusion, our report describes a new patient with a ring chromosome 2 completely characterised by array-CGH proving additional information useful not only to study genotype-phenotype correlation but also to validate the role of already reported candidate genes and to suggest novel ones which could improve our understanding of the clinical features associated with ring chromosome 2.

## Materials and methods

### Cytogenetic and CGH analyses

Standard GTG banding was performed at a resolution of 400–550 bands on metaphase chromosomes from peripheral blood lymphocytes of the patient and his parents. FISH analysis was performed using Aquarius Subtelomere Specific Probe 2p (D2S2983) (Spectrum Green)/2q (D2S2986) (Spectrum Orange) (Cytocell; http://www.cytocell.com). To better characterize the rearrangement, array-CGH analysis was performed using a 180 K platform with ~13 kb overall median probe spacing (Agilent Technologies, Santa Clara, CA). Labelling and hybridization were performed following the protocols provided by the manufacturers. A graphical overview was obtained using the Agilent Genomic Workbench Lite Edition Software 6.5.0.18.

### Brain MRI

Brain MRI was performed with a 1.5 T scanner (Achieva 2.6, Philips, Best, the Netherlands). DTI data were acquired using a single-shot spin-echo echoplanar sequence in 32 directions with TR/TE: 9318 ms/71 ms, FOV: 224 × 224 × 120 mm, flip angle: 90°, 60 slices, acquisition voxel size: 2 × 2 × 2 mm, slice thickness: 2 mm, slice gap: 0 mm, 2 b values of 0 and 800. 3D/TFE volumetric T1-weighted images were also acquired for registration. DTI data were analysed and DTT was performed using the fiber tract tool supplied by the manufacturer (Philips Extended Workspace, Philips, Best, The Netherlands).

## Consent

Written informed consent was obtained for publication and any accompanying images. A copy of the written consent is available.

## Additional file

Additional file 1: Table S1. MIM genes comprised in 2q37.3-qter deleted region.

## Abbreviations

IUGR: Intrauterine growth retardation
DTI: Diffusion tensor imaging
DTT: Diffusion tensor tractography
TFE: Turbo field echo
