# Supplementary material for: Clinico-radiological and molecular characterization of a child with ring chromosome 2 presenting growth failure, microcephaly, kidney and brain malformations
Source: Mol Cytogenet. 2015 Mar 5;8:17. doi: 10.1186/s13039-015-0121-z (PMC4359793; doi:10.1186/s13039-015-0121-z)
Supplement: Additional file 1: Table S1. — MIM genes comprised in 2q37.3-qter deleted region. [file 13039_2015_121_MOESM1_ESM.docx]

Table S1. MIM genes comprised in 2q37.3-qter deleted region

| Gene | Description | MIM number |
| --- | --- | --- |
| TWIST2 | Drosophila, homolog of, 2 | 607556 |
| HDAC4 | Histone deacetylase 4 | 605314 |
| NDUFA10 | NADH-ubiquinone oxidoreductase 1 alpha subcomplex, 10 | 603835 |
| OTOS | otospiralin | 607877 |
| GPC1 | Glypican 1 | 600395 |
| RNPEPL1 | Arginyl aminopeptidase-like 1 | 605287 |
| CAPN10 | Calpain 10 | 605286 |
| GPR35 | G protein-coupled receptor 35 | 602646 |
| AQP12A | Aquaporin 12A | 609789 |
| KIF1A | Kinesin family member 1A | 601255 |
| AGXT | Alanine-glyoxylate aminotransferase | 604285 |
| MTERFD2 | MTERF domain-containing protein 2 | 615393 |
| PASK | PAS domain-containing serine/threonine kinase | 607505 |
| PPP1R7 | Protein phosphatase 1, regulatory subunit 7 | 602877 |
| ANO7 | Anoctamin 7 | 605096 |
| HDLBP | High density lipoprotein-binding protein | 142695 |
| SEPT2 | Septin 2 | 601506 |
| FARP2 | FERM, RhoGEF and pleckstrin domain protein 2 | No MIM number  (HGNC: 16460) |
| STK25 | Serine/threonine protein kinase 25 | 602255 |
| BOK | BCL2-related ovarian killer | 605404 |
| THAP4 | THAP domain-containing protein 4 | 612533 |
| ATG4B | autophagy 4, s. cerevisiae, homolog of, b | 611338 |
| DTYMK | Deoxythymidylate kinase | 188345 |
| ING5 | Inhibitor of growth 5 | 608525 |
| D2HGDH | D-2-hydroxyglutarate dehydrogenase | 609186 |
| GAL3ST2 | Galactose-3-o-sulfotransferase 3 | 608234 |
| NEU4 | Neuraminidase 4 | 608527 |
| PDCD1 | Programmed cell death 1 | 600244 |
